# A 4-cm G2 cervical submucosal myoma removed with the IBS® Integrated Bigatti Shaver

**DOI:** 10.1007/s10397-012-0737-5

**Published:** 2012-03-03

**Authors:** G. Bigatti, C. Ferrario, M. Rosales, A. Baglioni, S. Bianchi

**Affiliations:** 1 U.O. di Ostetricia e Ginecologia, Ospedale Classificato San Giuseppe Via San Vittore, 12-20123 Milan, Italy; 2 Università degli Studi di Milano, Direttore dell’ Unità Opertiva di Ostetricia e Ginecologia, Ospedale Classificato San Giuseppe, Via San Vittore, 12-20123 Milan, Italy

**Keywords:** IBS®, Shaver, Operative hysteroscopy, New instrumentation

## Introduction

At present conventional bipolar resectoscopy is widely recognized as first choice procedure for major hysteroscopic operations [1, 2]. We have proposed an alternative approach to operative hysteroscopy called IBS® Integrated Bigatti Shaver [3] that improving the visualization during the procedure, as the tissue chips are removed from the uterine cavity at the same time of resection, reduces several problems of conventional resectoscopy such as fluid overload and water intoxication with a lower risk of uterine perforation. In cooperation with Karl Storz GmbH & Co., we have created a new shaving system that, introduced through a straight operative channel of a panoramic 90° optic, allows performing all kinds of major hysteroscopic operations. We have recently published the first randomized comparative study [4], designed in order to compare 50 cases performed with conventional bipolar resectoscope with 50 cases performed with the IBS®. All kinds of major intrauterine pathologies such as polyps up to 6 cm and G0, G1 and G2 sub-mucosal myomas, classified according to the ESGE guidelines, up to 3 cm in diameter were included in the study [5]. Dilatation time, global time of the procedure, resection time and fluid balance were carefully monitorized during each procedure of the two groups of patients.

The result of this study confirmed the several advantages offered by the IBS® such as better visualization during the procedure as tissue chips were removed at the same time of resection, no coagulation or cutting current was needed, utilization of normal saline and a much faster learning curve. In addition the 24 Fr total diameter of the optic allows a dilatation of the cervix up to number 8.5 of Hegar reducing the risk of cervical laceration and uterine perforation.

We could approach all kind of polyps and myomas up to 3 cm in a very fast, precise and clean way. The only drawback of this technique was represented by myomas with higher consistency and with a diameter larger than 3 cm. For this reason we have designed two new blades, number 6 which has a flute beak shape and number 7 which resemble the shark jaws in order to be more aggressive in cutting the fibrotic tissue. We report this interesting case in which the IBS® was able to remove in a two-step procedure a very large cervical G2 submucosal myoma, considered one of the most difficult cases to approach by conventional resectoscopy.

## Case presentation

A 37-year-old primiparous young patient came to our attention for menometrorrhagic cycles and intermenstrual bleeding. A transvaginal ultrasound was performed showing a posterior intracervical submucosal myoma of more than 4 cm in diameter. Diagnostic hysteroscopy confirmed the diagnosis, and we were able to assess that the largest part of the myoma was intramural and therefore classify the myoma as a G2 type.

**Fig. 1:**
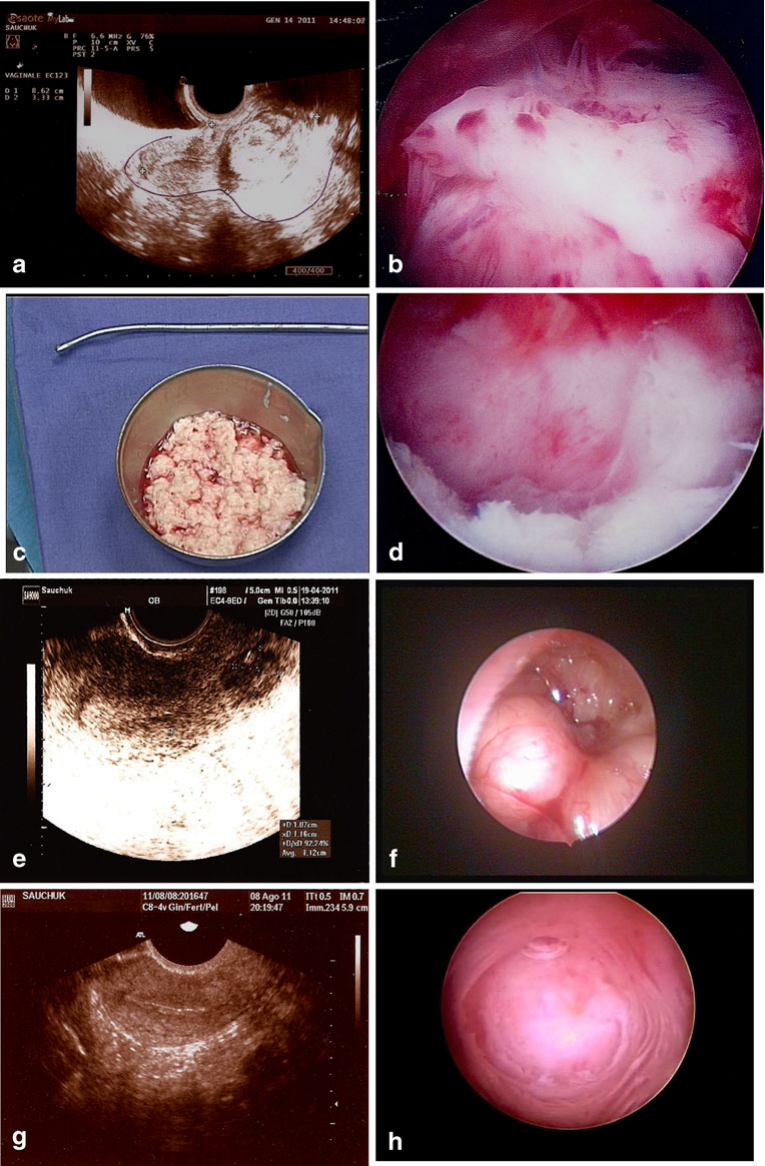
Primary procedure: **a** ultrasound of the 4 cm cubmucosal G2 myoma, **b** hysteroscopic view before the primary procedure, **c** 50 cc of tissue resected and **d** hysteroscopic view after the primary procedure. **e** Ultrasound 2 months after the primary procedure showing a 1-cm residual G1 myoma, **f** hysteroscopic view. **g** Ultrasound at 2 month after the secondary procedure showing a perfect cavity, **h** hysteroscopic view

A first operative hysteroscopy with the IBS® was planned. The whole procedure lasted 52 min with a resection time, which is the time effectively dedicated to remove the tissue, of 31 min. We removed three fourths of the myoma with a total of 12.500 ml of normal saline used and a fluid deficit of almost 1,000 ml. We stopped the procedure for safety reasons due to the particular position of the myoma as the intramural part of the myoma was compressing the serosal surface, impairing our capability to assess the safety margin of resection. No GNRH therapy was given prior to the intervention. We dilated the cervical canal until 8.5 of Hegar for a length of no more than 1.5 cm approximately as the myoma was placed very close to the external ostia of the cervical canal. We used a speed of resection of 450 rotation power per minute with an aspiration of 500 ml of fluid per minute. Blade number 7 was activated at the beginning of the procedure, and we switched to blade number 6, after approximately 15′, in order to test the different cutting power. Both blades were very good in removing the tissue, and 50 cc of myomatous tissue was sent to the pathologist for histological examination. The patient was discharged from hospital the day after the operation, and no complication occurred. Histology confirmed the diagnosis of submucosal leiomyoma. A transvaginal ultrasound was planned at 2 months after the surgical procedure. Despite a dramatic improvement of the symptomatology, the ultrasound scan showed a residual myoma of approximately 1 cm in diameter. The diagnosis was confirmed at hysteroscopy with an almost intracavitary position of the myoma (G1 type). A second operative procedure was planned with the IBS®, and the final operation was performed after 3 months. The second procedure lasted 19 min with a resection time of 15 min. We used 6,000 ml of normal saline with a fluid deficit of 500 ml approximately. A perfect fovea was left at the end of the procedure with a complete removal of the myomatous tissue. No complication was reported, and the patient was discharged from the hospital during the same day of the operation. A second ultrasound, performed after 1 month, showed a perfect healing of the uterine cavity with no remaining sign of the myoma (Fig. 1).

## Discussion

This case report confirms our thesis that almost all major hysteroscopic surgical procedures can be performed with the IBS® in a very easy, fast, precise and safe way. Especially for the treatment of large myomas up to 3 cm, it has several well-described advantages. As discussed by Emanuel et al., the diameter of an intrauterine pathology is strongly related to the operation time and to the complication rate [6]. Considering the volume calculation of the tissue to remove by the formula 4/3*π*r^3^, we need 8.4 min to resect 2 cm, 28.2 min for 3 cm and 67.0 min for 4 cm at a resection speed of 0.5 cm^3^/min for a conventional monopolar loop. Also in this case, the IBS® seems to be much faster than the conventional resectoscope. Probably this is due to the fact that the continuous cutting capacity performed always under direct visual control, with immediate removal of the chips at time of resection, results in a more efficient reduction of the tumour volume. Not only operation time but also total fluid loss seems to be better with the IBS® system. In fact the mean fluid deficit of the two procedures was limited to 750 ml with a mean of total fluid used of 9,250 ml. In addition the main advantage of the IBS® was that the myoma was effectively enucleated from its fovea and the intramural site of insertion of the myoma was removed. The surrounding healthy endometrium was avoided without any thermal injury occurring, compared with the less precise behaviour of conventional resectoscopy. No coagulation was needed, and there were no excess bleeding problems. At last, the approach of submucosal cervical myomas is considered one of the most difficult cases in operative hysteroscopy. The visibility during the procedure is reduced by the fact that the wall of the cervical canal blocks the holes of the aspiration system, placed at the lateral site of the outer sheet tip of the conventional resectoscope. The IBS® has a different aspiration system, directly from the front part of the tip, that always allows, especially in the treatment of intracervical pathologies, a better visualization. By the analysis of a small series of cases performed with blade number 6 and 7, which has to be confirmed by further studies, we found out that is not the size nor the sharpness of the blade but the consistency of the myoma the parameter that has to be considered in order to improve our cutting capability. We believe that the success of this case was also due to the reduced consistency of this myoma associated with a higher cutting power of our blades.

For very large myomas, we will possibly need to improve our blade system because at present the IBS® has still some drawbacks. Compared to other blind intrauterine applications, the IBS® has the major advantage that surgeons always perform the procedure under visual control, with automated and easy removal of tissue chips. Very interesting is that the IBS® is able to resect also myomatous tissue, making this a very promising alternative to the resectoscope. In fact surgery is not interrupted by tissue chips removal, making total operating time shorter. It is further postulated that resection of myomas without the use of electrical current could significantly reduce the postoperative adhesion formation and that the IBS® should preferentially be used in younger women in their reproductive age [7].

## Conclusion

We believe that the success in removing such a large cervical myoma with the IBS® supports our thesis that we are working in the right direction. Certainly the IBS® is at the beginning of its history and can only be improved in its functionality. At present we are testing the possibility of a bipolar coagulation system through the operative channel of the optic in order to prevent possible bleeding in the treatment of large myomas. Anyway the mechanical removal of pathological tissues from the uterine cavity without the use of current makes the procedure much safer. Further studies are planned in order to confirm the real value of the IBS® in the treatment of submucosal myomas.
